# Finance

**Published:** 1892-08-06

**Authors:** Richard Kershaw

**Affiliations:** Secretary of the Central London Throat and Ear Hospital


### HOSPITAL OPINION.

[Contributions critical, suggestive, controversial, and technical are invited for this column, which is open to everyone who has anything to say that is of practical value and interest.]

### FINANCE.

Mr. Richard Kershaw, Secretary of the Central London Throat and Ear Hospital, writes: The fallacy of such a method of calculating hospital expenditure as that suggested in your article under the above heading cannot better be shown than by a *reductio ad absurdum.* To take the case of a hospital similarly situated as this one in all respects save that it contains but four beds as against our sixteen, the expenditure of that hospital is relatively the same as our own. Will it be contended that its total expenditure should be divided by the number of beds in daily occupation? If so, you would get an average cost of £500 per bed, and while the result, according to the above method, might be strictly accurate as calculated on the number of beds occupied, nothing could be more misleading to those who are not thoroughly conversant with the compilation of hospital statistics. In point of fact, the "average cost per occupied bed" is no criterion whatever of the economic management of any hospital if it is arrived at in the manner illustrated in your article, for it is practically impossible to compare things dissimilar. In a large general hospital the main portion of the expenditure pertains to the in-patient department, and a few thousand more or less out-patients makes no material difference in the cost; but in a small or special hospital with few beds, but with a large out-patient department, the greater portion of the expenditure is that incurred by the out-patients. Thus at this hospital the total cost of 6,450 out-patients amounted to £1,217, and that for the in-patients to £762, which proves the "average cost of each occupied bed" to be £63 5s. 4d., and the average cost of each out-patient to be 3s. 9d. This was stated in our return to the Hospital Sunday Fund, from the statistics of which, provided for the use of the Council, your correspondent has evidently taken his figures. See also Appendix E, Report of the Select Committee on Metropolitan Hospitals, August, 1890. The witness from this hospital in his evidence before the Lords' Committee, drew attention to the injustice of the Charity Organisation Society in arbitrarily assessing the cost of each out-patient at one shilling. Your correspondent has fairly capped this by ignoring our out-patients altogether.

[In estimating the average cost of a bed at his hospital, Mr. Kershaw is not far wrong in his contention that a special hospital which can only boast of twelve beds in continuous occupation must not be judged by the standard applicable to larger institutions. At the same time, it is a mistake to dignify such establishments with the name of hospitals, when they are neither more nor less than dispensaries with a few beds added, and are restricted to giving advice and medicine at the central bureau. Whether the 6,450 out-patients Mr. Kershaw speaks of would not be equally well treated at the general hospitals is another question, but granting that the average cost of the twelve beds may, by an arithmetical puzzle, be reduced from £164 to £63 a-piece, surely the estimated expense of the out-patients (3s. 9d. a-head) is excessive when compared with the slight outlay of the department. Our correspondent admits that the high cost of some of the special hospitals, when estimated by the standard adopted, is attributable to the paucity of their accommodation, but this might be simplified and corrected by their being placed in the category of dispensaries. All these smaller institutions ought to publish separate accounts of the expenditure (*a*) on in-patients, and (*b*) on out-patients only.—Ed. *T. H.*]

